# Management of jejunoileal atresias: an experience at eastern Nepal

**DOI:** 10.1186/1471-2482-10-35

**Published:** 2010-11-26

**Authors:** Vikal C Shakya, Chandra S Agrawal, Pramod Shrestha, Prakash Poudel, Sudeep Khaniya, Shailesh Adhikary

**Affiliations:** 1 Department of Surgery, B. P. Koirala Institute of Health Sciences, Dharan, Nepal; 2 Department of Pediatrics, B. P. Koirala Institute of Health Sciences, Dharan, Nepal

## Abstract

**Background:**

Intestinal atresia is a common cause of neonatal intestinal obstruction, and management of this disease in limited setup of a developing country is very difficult.

**Methods:**

This study is a retrospective study of patients with jejunoileal atresias and their postoperative outcome in a teaching hospital in eastern Nepal over a 5-year period.

**Results:**

There were 28 children (19 boys and 9 girls). 11 children (39.28%) had jejunal atresia and 17 (60.71%) had ileal atresia. Eight (28.5%) patients died, 6 were jejunal atresia (54.5%) and 2 were ileal atresia (11.7%). The most common cause of death was sepsis which occurred in 7 out of 8 cases (87.5%). The risk factors for mortality identified were leucopenia, neutropenia, delay in surgery, location of atresia and type of atresia. Jejunal atresia tended to have a higher mortality than ileal atresia, and severe types of atresia (type IIIb and IV) were more often associated with mortality than other types of atresia. The significant differences between jejunal and ileal atresia were the increased duration between presentation and surgery, longer postoperative and total hospital stay, presence of more severe atresias and an increased risk of mortality in case of jejunal atresias.

**Conclusion:**

The prognosis for this disease have definitely changed in the last few decades in developed countries but in our environment, problems like late presentation and diagnosis, lack of availability of good neonatal intensive care units and parenteral nutritional support still prevail.

## Background

Intestinal atresia is a common cause of neonatal intestinal obstruction. In last few decades, the management of neonates with jejunoileal atresias has improved because of improvements neonatal intensive care, advances in operative techniques and use of total parenteral nutrition (TPN)[1-3]. With these approaches, the morbidity and mortality of these patients has decreased to a large extent. However, the scenario in developing countries like Nepal is still different; we have our own inherent problems in managing these children in our limited setup, and mortality remains high as in other underprivileged countries[4-6]. This study is a retrospective study to analyze clinical characteristics in patients with jejunoileal atresias and their postoperative outcome in a teaching hospital in eastern Nepal over a 5-year period and we intend to highlight the management problems in our environment.

## Methods

From January 2004 to December 2008, 28 children with jejunoileal atresia were operatively managed at the Department of Surgery, B. P. Koirala Institute of health Sciences situated at eastern Nepal. This study is a retrospective hospital-based study of these children, we reviewed their case notes, operation notes, and discharge summary and analyzed their outcome.

The babies were resuscitated with intravenous fluids, nasogastric decompression was done, urethral catheter was placed, and intravenous antibiotics (cefotaxime and gentamicin) and metronidazole were given. They were planned for laparotomy after informed consent by the parents. During operation, minimal resection of the proximal bowel with antimesenteric tapering enteroplasty and end-to-end anastomosis with 4-0 vicryl had been for all jejunal atresias and proximal ileal atresias. Transanastomotic tubes had not been used. In patients with distal ileal atresia, ileostomy with distal mucous fistula was done. TPN could not be offered to these patients after surgery, due to its unavailability in our region. Postoperatively, the babies were kept nil per oral alongwith nasogastric decompression for a few days, thereafter, the babies were fed with expressed breast milk via the infant-feeding tube when baby passed stool and the gastric aspirate was minimal. When active, the babies were allowed to suck the mother's breast.

Data analysis was performed using the SPSS software package (version 15.0, SPSS Inc, Chicago, IL). Data were analyzed using the X^2 ^test for categorical data and student's t-test for continuous variables. Two-tailed values of P below 0.05 were considered statistically significant. Data are summarized using mean and standard deviation for normally distributed variables and median for non-normally distributed continuous variables. All parents signed appropriate informed consent for their inclusion in the study. The current study was approved by the hospital ethical committee. We also attest that this human study undertaken as part of the research from which this manuscript was derived is in compliance with the Helsinki Declaration.

## Results

There were a total of 28 children which included 19 boys and 9 girls. The median age at presentation was 3 days (range 1 day - 18 months). Two children presented late by virtue of partial obstruction; one with jejunal web presented at 18 months and another with ileal web came at 42 days of age. The median weight at presentation was 2.7 kg (range 2-4 kg). Five patients were preterm (history regarding exact gestational age could not be elicited in most children; majority of mothers could tell only whether the child was term or preterm). Two mothers (7.1%) had history of polyhydramnios. Only 3(10.71%) children were born in the same hospital, 10(35.71%) were delivered in other hospitals and 15(53.57%) were born at home. Bilious vomiting and abdominal distension were present in all (100%) patients, jaundice was present in 5 (17.8%) patients and fever was present in 4 (14.2%) patients. Five patients were suspected initially to have neonatal septicemia with septic ileus and were managed in the pediatric ward, which later turned out to be atresias. Three patients (10.7%) passed meconium prior to presentation (a jejunal web, an ileal web, and another atresia at the duodenojejunal flexure). These children had appreciable dilatation of the rectum. Rest of the children did not pass meconium and the rectum negotiated only an infant feeding tube. Plain abdominal x-ray was done in all 28 children. Two patients with jejunoileal atresias had a barium enema done which showed a microcolon. Two children (7.1%) presented with pneumoperitoneum.

11 children (39.28%) had jejunal atresia and 17 (60.71%) had ileal atresia. One patient had a jejunal web, which was excised after an enterotomy, and a Heineckie-Meckuliz enteroplasty was done. Another patient had an ileal web, which was excised with closure of the enterotomy. A significant finding in these two patients that differ from others is that these webs were incomplete, they were passing stools at presentation and presented late. All jejunal atresias and 7 patients with proximal ileal atresias underwent primary anastomosis. In one patient with combined proximal ileal and colonic atresia, excision of the atretic segment alongwith ileotransverse anastomosis was done. In other 9 patients with distal ileal atresia, ileostomy with distal mucous fistula was done. In 6 patients ileostomy closure has been done, the remaining 3 patients are planned for ileostomy closure. Two neonates had associated malrotation, one had distal ileal volvulus, one patient had jejunal atresia associated with an enteric duplication cyst with multiple true diverticuli, and another had ileal atresia due to entrapment in the falciform ligament[7]. Another patient had ileal atresia due to entrapment in the omphalic ring[8]. The babies were orally allowed after a mean postoperative period of 5.03±1.56 days (range 3-8 days); babies with jejunal atresias were orally allowed after 5.66±1.11 days (range 4-8 days), and those with ileal atresias were orally allowed after 4.7±1.68 days (range 3-8 days).

Sepsis occurred in 7 (25%) patients; 7 (25%), patients had superficial wound infections; three patients each (25%) had apnea and hyponatremia postoperatively; two patients (7.1%) each had burst abdomen, postoperative pneumonia and seizures; one patient each (3.5%) had peristomal cellulitis and parastomal herniation, which required redo operation in the form of a double-barrel ileostomy, and one patient (3.5%) had adhesive obstruction which presented after 1 month of initial surgery. Three patients (25%) had anastomotic dehiscence with intraabdominal abscess that required laparotomy for drainage. Eight (28.5%) patients died, 6 were jejunal atresia (54.5%) and 2 were ileal atresia (11.7%). The most common cause of death was sepsis which occurred in 7 out of 8 cases (87.5%). One patient with jejunal atresia died due to aspiration when started orally, two died from sepsis after intraabdominal abscess formation from an anastomotic leak and were reoperated, but could not be saved. Three patients died due to persistent abdominal distension due to paralytic ileus and sepsis. One patient with ileal atresia also died due to leak and sepsis, another one on stoma died due to sepsis, disseminated intravascular coagulation and gangrene of the stoma.

The risk factors for mortality identified were age at presentation, leucopenia, neutropenia, duration from presentation to surgery, location of atresia and type of atresia (Table 1, 2). Though overall delay in presentation has not been found to be a significant factor for mortality (p-value 0.85), two of the patients had incomplete webs, which led to their late presentation. If we evaluate only those children who had complete obstruction, then delay in presentation still becomes a significant factor for mortality (p-value 0.04). The mean TLC in survivors was 15304±12835.71 per ml, whereas in non-survivors, it was 12138±4933.25per ml (p-value 0.01). Non-survivors tended to have a significantly lower neutrophilia than survivors (47% vs. 51%) (p-value 0.02). Delay in operation was also a significant factor for survival (23.55±33.5 hrs for survivors, and 48.37±88.14 hrs for non-survivors) (p-value 0.0007). Jejunal atresia tended to have a higher mortality than ileal atresia (p-value 0.01), and the severe types of atresia (type IIIb and IV) were more often associated with mortality than other types of atresia (p-value 0.006).

**Table 1:** Type of atresia associated with location and survival

Type of atresia	Location		Survival	
	Jejunal(n = 11)	Ileal(n = 17)	Survivors(n = 20)	Non-survivors(n = 8)
I	1	1	2	0
II	1	1	0	2
IIIa	2	12	14	0
IIIb	4	1	2	3
IV	3	2	2	3
Total	11	17	20	8

**Table 2:** Risk factors for mortality

Type of atresia	Survivors (n = 20)	Non-survivors (n = 8)	p-value
Age at presentation (days)	33.2±119.64	6.33±7.06	0.85
	4.55±3.97*	6.33±7.06*	0.04*
Weight at presentation (kg)	2.95±0.54	2.53±0.4	0.26
Total leucocyte count (per ml)	15304±12835.71	12138±4933.25	0.01
Differential neutrophils count	59±11.47%	47±21.63%	0.02
Duration between presentation and surgery (hrs)	23.55±33.5	48.37±88.14	0.0007
Jejunal atresia	5(25%)	6(75%)	0.01
Severe atresias	4(20%)	6(75%)	0.006

*This doesn't include values for patients with incomplete webs who presented late, which affected the previous value

The characteristics of jejunal and ileal atresias in this study were also different in many aspects (Table 3). The significant differences between jejunal and ileal atresia were the increased duration between presentation and surgery (p-value 0.02), longer postoperative (p-value 0.01) and total hospital stay (p-value 0.001), presence of more severe atresias (p-value 0.01) and an increased risk of mortality in cases of jejunal atresias (p-value 0.001). There was no significant difference in the two groups in the sex ratio (p-value 0.22), age at presentation (p-value 0.1) and birth weight (p-value 0.69), and frequency of complications (p-value 0.54).

**Table 3:** Differences between jejunal and ileal atresias

Type of atresia	Jejunal(n = 11)	Ileal(n = 17)	p-value
Sex (Male/Female)	1.2:1	3.25:1	0.22
Duration of presentation (hrs)	54.18±161.24	7.11±9.89	0.1
Birth weight (kg)	2.62±0.46	2.69±0.51	0.69
Duration between presentation and surgery (hrs)	27.45±39.32	17.41±20.82	0.02
Postoperative hospital stay (days)	10.28±4.73	8.76±2.33	0.01
Total hospital stay (days)	13.09±6.64	10.11±2.73	0.001
Severe atresias	7 (63.63%)	3 (17%)	0.01
Complications	8	14	0.54
Mortality	6 (54.54%)	2 (11.76%)	0.001

## Discussion

Intestinal atresia is a common cause of neonatal intestinal obstruction. Vascular accidents are thought to predispose to a majority of these lesions[9]. There have been encouraging recent reports in management of intestinal atresia. However, the situation in developing countries has not changed. This study is intended to review the clinical characteristics and management of 28 children with jejunoileal atresias presenting to an eastern region hospital in Nepal, a South Asian country.

The clinical features of these children deserve special consideration. Failure to pass meconium was another common presenting symptom, however, it was absent in cases of incomplete ileal and jejunal webs and a case of atresia at the duodenojejunal flexure. In this report, the median age at presentation was 3 days. One of the reasons that we found for the delay in presentation was the surprising misinterpretation of passage of mucus as meconium by the parents and even by the peripheral health workers. None of our patients were diagnosed in the antenatal period. Antenatal diagnosis could have decreased the duration of presentation to our hospital. As already mentioned, majority of our deliveries still occur at home means that delay in presentation is inevitable, which was found to be a significant factor for mortality. Apart from delayed presentation, there were delays in operative intervention also due to diagnostic dilemmas, especially where the child passed meconium before presentation. Even in cases of complete obstructions, diagnosis was delayed due to predominant presentation in the form of neonatal sepsis with jaundice, lethargy and fever. Perhaps a policy of using early barium enema in these suspected children could have reduced this duration and subsequent adverse outcome.

Another aspect that should be mentioned is the bitter fact that the neonatal intensive care unit of our hospital is usually filled with large volume of infectious illnesses by virtue of our tropical location, so only few of our children could be treated in the intensive care unit. Though statistical analysis could not be done, this could have been a significant factor in the outcome of the disease. Management strategies had to be modified therefore, considering the clinical status of the patients and the deficiencies in investigative and management facilities, especially the unavailability of TPN, elemental feeds and intensive care facilities. We preferred for tapering enteroplasty and anastomosis after minimal resection for jejunal and proximal ileal cases to avoid the consequences of short bowel syndrome, which could never have been managed in our setting[10]. The distal ileal atresias were managed by stomas because a greater number of anastomosis could have meant a higher chance of leaks in these sick babies and subsequent dismal prognosis, and a greater number of stomas would have necessitated the use of TPN, which is not possible in our set-up. The postoperative mortality of 28.5% in this series differs largely from data in the developed countries, but it is comparable to with reports from the third world[1-6]. Kumaran et al reported 10% mortality rate at England[2], Stollman et al reported a mortality rate of 11% at Netherlands[3] whereas Chirdan et al reported mortality of 41.7% in Nigeria[6], a figure much like in our study. The increased mortality in our study could be attributed to minimal resection of the dilated proximal bowel and resultant hypotonic functional obstruction and abnormal motility in the retained dilated proximal bowel[1], and mortality has been found to be decreased by generous resection of the proximal bowel and anastomosis[2-10], but it would have increased the incidence of short bowel syndrome, which we wished to avoid, because it could never have been managed in our setting without TPN. We had a high incidence of early morbidities than other studies, which were tackled as far as possible.

The risk factors for mortality identified were leucopenia, neutropenia, delay in presentation, duration from presentation to surgery, location of atresia and type of atresia. Leucopenia and neutropenia are factors that have never been identified before. Probably those patients in whom the immunity is not good enough to respond to the hostile environment do not fare well to survive. As has been mentioned, duration from presentation to surgery was another risk factor. The longer the time taken to present to the hospital, for diagnosis, resuscitation and intervention was, the graver the prognosis would be. Mortality was also found to be associated with location and types of atresia. The more distal the lesion the more are the chances of survival. Severe atresias (type IIIb and IV) had a significantly higher mortality than other types of atresia. Burjonruppa et al has also identified severe atresias as significant predictors of mortality[11]. Jejunal atresias were of more severe forms than ileal ones (63.63% of jejunal atresias were type IIIb or IV, whereas only 17% of ileal atresias were type IIIb or IV). The higher mortality in jejunal atresia was probably due to presence of other congenital anomalies which were missed. Though none of our patients had a clinically detectable congenital anomaly, these might have been a significant factor in the outcome. Rigorous search for associated anomalies was not feasible in our study; another reason that these were not picked up may be small volume of cases. We also found location of atresia as significant factor for survival. Ileal atresias had a significantly better survival than jejunal atresia, like in study by Tongsin et al[12]. In some earlier studies, jejunal atresia fared better, whereas in others, there was no association[11,13]. Unlike other studies, we did not find significance of weight in relation to mortality[12,13].

The prognosis for intestinal atresia has improved significantly in recent years because of development in procedures for preservation of intestinal length, better management of associated anomalies, small bowel transplantation and improved perioperative management. In developing countries however, as in our environment, the situation is different and the results are still far from good. Late presentation and/or diagnosis resulting in deterioration of the patient, lack of availability of neonatal intensive care units due to occupancy by non-surgical diseases and lack of parenteral nutritional support are bitter factors that definitely account for the poor outcome in our setup. We accept with our heart that our results do not conform to international standards, but there are very few options presently. Probably the proportions of death in our study could have been decreased by extensive search for associated congenital anomalies, the availability of TPN and better intensive care facilities, which we hope to achieve in the near future.

## Conclusion

The prognosis for intestinal atresia in the last few decades may have changed in developed countries but our environment still suffers from problems like late presentation and diagnosis, lack of availability of good neonatal intensive care units and parenteral nutritional support which may be responsible for the poor outcome in our setup.

## Abbreviations

TPN: Total parenteral nutrition; TLC: Total leucocyte count.

## Competing interests

The authors declare that they have no competing interests.

## Authors' contributions

VCS, SK, PP and PS made substantial contributions to concept and design of the article. CSA and SA contributed significantly in critical revision and drafting the manuscript. All authors read and approved the final version of the manuscript

## Pre-publication history

The pre-publication history for this paper can be accessed here:

http://www.biomedcentral.com/1471-2482/10/35/prepub
